# A multiple-behaviour investigation of goal prioritisation in physicians receiving audit and feedback to address high-risk prescribing in nursing homes

**DOI:** 10.1186/s43058-020-00019-3

**Published:** 2020-02-25

**Authors:** Nicola McCleary, Laura Desveaux, Catherine Reis, Stefanie Linklater, Holly O. Witteman, Monica Taljaard, Kednapa Thavorn, Jeremy M. Grimshaw, Noah M. Ivers, Justin Presseau

**Affiliations:** 1 Centre for Implementation Research, Clinical Epidemiology Program, Ottawa Hospital Research Institute, The Ottawa Hospital - General Campus, 501 Smyth Road, Room L1202, Box 711, Ottawa, ON K1H 8L6 Canada; 2 School of Epidemiology and Public Health, University of Ottawa, Ottawa, Canada; 3 Women’s College Research Institute, Women’s College Hospital, Toronto, Canada; 4 Women’s College Hospital Institute for Health System Solutions and Virtual Care, Women’s College Hospital, Toronto, Canada; 5 Institute of Health Policy, Management and Evaluation, University of Toronto, Toronto, Canada; 6 Department of Family and Emergency Medicine, and Office of Education and Continuing Professional Development, Laval University, Québec City, Canada; 7 Laval University Research Institute for Primary Care and Health Services, Laval University, Québec City, Canada; 8 Department of Medicine, University of Ottawa, Ottawa, Canada; 9 Department of Family and Community Medicine, University of Toronto, Toronto, Canada; 10 ICES, Toronto, Canada; 11 School of Psychology, University of Ottawa, Ottawa, Canada

**Keywords:** Goal priority, Healthcare provider behaviour, Nursing home, Audit and feedback, Prescribing, High-risk medications

## Abstract

**Background:**

As part of their professional role, healthcare providers enact multiple competing goal-directed behaviours in time-constrained environments. Better understanding healthcare providers’ motivation to engage in the pursuit of particular goals may help inform the development of implementation interventions. We investigated healthcare providers’ pursuit of multiple goals as part of a trial evaluating the effectiveness of an audit and feedback intervention in supporting appropriate adjustment of high-risk medication prescribing by physicians working in nursing homes. Our objectives were to determine whether goal priority and constructs from Social Cognitive Theory (self-efficacy, outcome expectations, and descriptive norms) predicted intention to adjust prescribing of multiple high-risk medications and to investigate how physicians in nursing homes prioritise their goals related to high-risk medication prescribing.

**Methods:**

Physicians in Ontario, Canada, who signed up for and accessed the audit and feedback report were invited to complete a questionnaire assessing goal priority, self-efficacy, outcome expectations, descriptive norms, and intention in relation to the three targeted behaviours (adjusting prescribing of antipsychotics, benzodiazepines, and antidepressants) and a control behaviour (adjusting statin prescribing). We conducted multiple linear regression analyses to identify predictors of intention. We also conducted semi-structured qualitative interviews to investigate how physicians in nursing homes prioritise their goals in relation to appropriately adjusting prescribing of the medications included in the report: analysis was informed by the framework analysis method.

**Results:**

Thirty-three of 89 (37%) physicians completed the questionnaire. Goal priority was the only significant predictor of intention for each medication type; the greater a priority it was for physicians to appropriately adjust their prescribing, the stronger was their intention to do so. Across five interviews, physicians reported prioritising adjustment of antipsychotic prescribing specifically. This was influenced by negative media coverage of antipsychotic prescribing in nursing homes, the provincial government’s mandate to address antipsychotic prescribing, and by the deprescribing initiatives or best practice routines in place in their nursing homes.

**Conclusions:**

Goal priority predicted nursing home physicians’ intention to adjust prescribing. Targeting goal priority through implementation interventions therefore has the potential to influence behaviour via increased motivation. Implementation intervention developers should consider the external factors that may drive physicians’ prioritization.

Contributions to the literature

- Time constraints introduce competition amongst healthcare providers’ multiple goals.
- After receiving audit and feedback, providers must prioritise their goals for practice change: how they do this is not fully understood.
- We found that the priority of a goal predicted nursing home physicians’ intention to adjust their prescribing of multiple high-risk medications after receipt of audit and feedback and that specific deprescribing goals were prioritised based on government policy, media coverage, and facility-level initiatives/routines.
- Our focus on multiple prescribing behaviours adds to recognised gaps in our understanding of how providers prioritise their goals in response to an implementation intervention.

## Background

Individuals have and pursue multiple goals, where goals are conceptualised as the aims or objectives that an individual is trying to reach as a result of their behaviour [1]. Healthcare providers perform multiple behaviours as part of their professional role; however, evidence indicates that time constraints introduce competition amongst the multiple goals, and this influences which goals are achieved [2]. Better understanding healthcare providers’ motivation to engage in the pursuit of particular goals may help inform the development of implementation interventions designed to address gaps between optimal quality care as determined by best current evidence and the care that patients actually receive [3].

We investigated healthcare providers’ pursuit of multiple goals as part of a trial evaluating the effectiveness of an audit and feedback (A&F) intervention. A&F involves measuring a provider’s practice, comparing it to a benchmark, and providing this information back to the provider to encourage change where appropriate [4]. A Cochrane review of 140 randomised trials found that A&F leads to a median 4.3% absolute improvement (interquartile range 0.5% to 16%) in the provision of recommended care [4]. Reported use of behavioural theory in A&F research is rare, with only 9% of studies reporting the use of theory to inform intervention development [5]. Increasing the appropriate use of theory in A&F research has been recommended [6] and could help researchers establish mechanism(s) of effect through the examination of underlying behavioural processes and inform the iterative development of subsequent interventions.

We are evaluating the effectiveness of A&F in supporting appropriate adjustment of high-risk medication prescribing by physicians working in nursing homes [7]. Medications such as antipsychotics and benzodiazepines are known to pose substantial risks in older people and may be relatively overprescribed given the risk/benefit ratio [8–13]. Based on Social Cognitive Theory [14, 15], A&F was hypothesised to influence prescribing behaviour primarily by increasing physicians’ intentions to appropriately adjust their prescribing (conceptualised within this theory as proximal goals). Intention is theorised to be a proximal predictor of behaviour, and meta-analyses indicate that intention is a consistent predictor of health behaviour in patients and the public [16, 17]. In addition, interventions which lead to medium-to-large increases in intention are also likely to lead to small-to-medium changes in behaviour [17]. Further evidence suggests that the relationship between intention and behaviour typically seen when predicting health behaviours also holds for healthcare provider behaviours [18, 19]. Thus, intention is a key mechanism of behaviour change which can be targeted with implementation interventions. In line with our theoretical basis, increases in intention due to the A&F were hypothesised to occur via increases in self-efficacy (an individual’s confidence in their ability to perform the behaviour), outcome expectations (beliefs about outcomes that may result from performance of the behaviour), and descriptive norms (beliefs about others’ performance of the specific behaviour).

However, this does not fully account for the multiple behaviour perspective incorporated in the trial through the provision of feedback on multiple prescribing indicators. After receipt of feedback relating to many of their behaviours, providers likely must prioritise any practice change efforts that they deem to be appropriate. Previous research shows that healthcare providers’ behaviour in the context of multiple goals may be predicted in part by how those goals are perceived to compete with or facilitate each other [2, 20]. Although previous research has focused on goal facilitation and goal conflict, we are not aware of any work investigating how healthcare providers prioritise the pursuit of goals.

In addition, health behaviour theories tend to focus on understanding a single behaviour in isolation from others, and as a result, most health behaviour studies tend to take a single-behaviour focus [21–23]. However, Conner et al. [24] conducted a series of studies to investigate the influence of goal priority and goal conflict on the relationship between intention and health behaviour (e.g. conducting vigorous physical activity). Although goal priority and goal conflict were each associated with both intention and behaviour, only goal priority moderated the intention-behaviour relationship, whereby the higher the priority of the goal, the stronger the prediction of behaviour by intention [24]. Further investigation of this across a wide range of behaviours is required. We therefore planned to explore goal prioritization in the context of this A&F trial. The objectives were to determine whether goal priority, self-efficacy, outcome expectations, and descriptive norms were predictors of intention to appropriately adjust prescribing of multiple high-risk medications and to investigate how physicians in nursing homes prioritise their goals generally and specifically in relation to appropriately adjusting prescribing of the high-risk medications included in the A&F.

## Methods

### Design and setting

This mixed-methods study was embedded within a 2 × 2 factorial, cluster-randomised trial conducted through the Ontario Health Implementation Laboratory (OHIL), a partnership between Women’s College Hospital (WCH), the Ottawa Hospital Research Institute (OHRI), and Health Quality Ontario (HQO; the provincial advisor on quality in healthcare in Ontario, Canada). The trial focuses on HQO’s ‘*MyPractice*: Long-Term Care’ report (http://www.hqontario.ca/Quality-Improvement/Guides-Tools-and-Practice-Reports/Long-Term-Care) and is testing the impact of variations in (i) the comparator and (ii) feedback message framing, on the effectiveness of A&F to reduce prescribing of high-risk medications. The feedback focuses on the potential increased risk of falls related to medication use. Further details of the trial methods and A&F variants being evaluated are provided in the protocol [7]. A theory-based mixed-methods process evaluation [25, 26] was conducted alongside the trial, using structured questionnaires followed by one-on-one semi-structured qualitative interviews. This enabled us to investigate the motivational constructs within the study reported here. A mixed-methods approach was necessary for answering our research questions, which focused on assessment of relationships between variables and on assessment of physicians’ views. Our methods are reported in accordance with the Good Reporting of A Mixed Methods Study (GRAMMS) Checklist (Additional file 1) [27].

### Quantitative data collection and analysis

#### Participants and recruitment

Physicians in Ontario, Canada, who voluntarily signed up to and accessed their A&F report were sent an email invitation to complete a structured questionnaire.

#### Materials and procedure

The questionnaire assessed the theoretical constructs targeted by the A&F: intention, self-efficacy, outcome expectations, descriptive norms, and goal priority (Table 1). Questions were based on three behaviours related to prescribing of medications focused on in the report (antipsychotics, benzodiazepines, and antidepressants) and a fourth behaviour related to prescribing of statins, included as a ‘control behaviour’ to allow us to observe trends in theoretical constructs. A single question was included to measure each theoretical construct for each behaviour. Each item was scored by participants using a 5-point Likert scale, with high scores representing agreement and low scores disagreement.

**Table 1:** Theoretical constructs investigated

Construct	Question
Intention	Regarding prescribing antipsychotics^a^ for my residents in my long-term care facility over the next month… I intend to appropriately adjust my prescribing for antipsychotics^a^
Self-efficacy	Regarding prescribing antipsychotics^a^ for my residents in my long-term care facility over the next month… given the features of my LTC facility, I am confident that I can appropriately adjust my prescribing for antipsychotics^a^
Outcome expectation	Regarding prescribing antipsychotics^a^ for my residents in my long-term care facility over the next month… I will avoid unnecessary risks to my residents’ health if I appropriately adjust my prescribing for antipsychotics^a^
Descriptive norm	Regarding prescribing antipsychotics^a^ for my residents in my long-term care facility over the next month… my colleagues in other LTC homes in Ontario are appropriately adjusting their prescribing for antipsychotics^a^
Goal priority	Regarding prescribing antipsychotics^a^ for my residents in my long-term care facility over the next month… it is a priority for me to appropriately adjust my prescribing for antipsychotics^a^

*LTC* long-term care

^a^Or benzodiazepines, or antidepressants (SSRIs (selective serotonin reuptake inhibitors) including trazodone and TCAs (tricyclic antidepressants)), or statins

#### Data analysis

For the current study, data from all trial groups were combined to explore the relationships between hypothesised predictor constructs and intention to appropriately adjust prescribing, controlling for each intervention factor as a covariate. In line with theory and research described above [14, 24], we used multiple linear regression to explore the relationships between hypothesised predictor constructs (self-efficacy, outcome expectations, descriptive norms, and goal priority) and intention to appropriately adjust prescribing, while controlling for each of the two intervention factors as covariates. Analyses were conducted using SPSS 24.

### Qualitative data collection and analysis

#### Participants and recruitment

The final questionnaire item allowed willing participants to self-identify for interview participation. Those indicating interest were invited by email to take part in a telephone interview. We aimed to apply the ‘10 + 3 rule’ to achieve thematic saturation, whereby ten interviews were to be conducted and analysed, followed by an additional three interviews. If these three interviews did not raise any new themes, we would take this as evidence of saturation [28]. Otherwise, we planned to proceed until no new themes were identified from three consecutive interviews.

#### Materials and procedure

A topic guide was developed, including questions specifically focused on goal prioritization in relation to prescribing behaviour change (included in Additional file 2). The topic guide was pilot tested and refined prior to full-scale use.

#### Data analysis

Interviews were digitally audio-recorded, then transcribed verbatim by an external third party. Transcripts were de-identified. The analytical method involved an iterative process of data collection and analysis informed by the framework analysis method [29, 30] and using qualitative software NVIVO 10. We deductively applied the theoretical constructs as codes, as well as constructs from the Consolidated Framework for Implementation Research (CFIR) [31] (to explore the contextual factors that interact with the A&F, to be described in the report of the trial results). Open coding was applied as required to allow us to incorporate themes not captured within these constructs. An initial coding framework was developed: two researchers (NMc and SL) independently applied it to the first two transcripts. The researchers met to discuss their coding and refine the framework and themes after each of the transcripts had been coded. The resulting framework was then applied to the remaining transcripts. Refinement of themes involved discussion with other team members (LD, CR, JP) and the wider study team as necessary and included reflection on the quantitative data.

## Results

### Quantitative questionnaire

#### Response rates and participant characteristics

Of the 267 physicians for whom HQO generated an A&F report, 89 downloaded their report and received an email invitation to complete the questionnaire; 33 (37.1%) participated. On average, participants had been providing care in nursing homes for 22.0 years (SD 13.0), had been practicing in their current nursing home for 17.2 years (SD 11.2), and spent a median of 1 day per week providing clinical care in their nursing home (IQR 0.9 to 2.0).

#### Predictors of intention to appropriately adjust prescribing

**Fig. 1:**
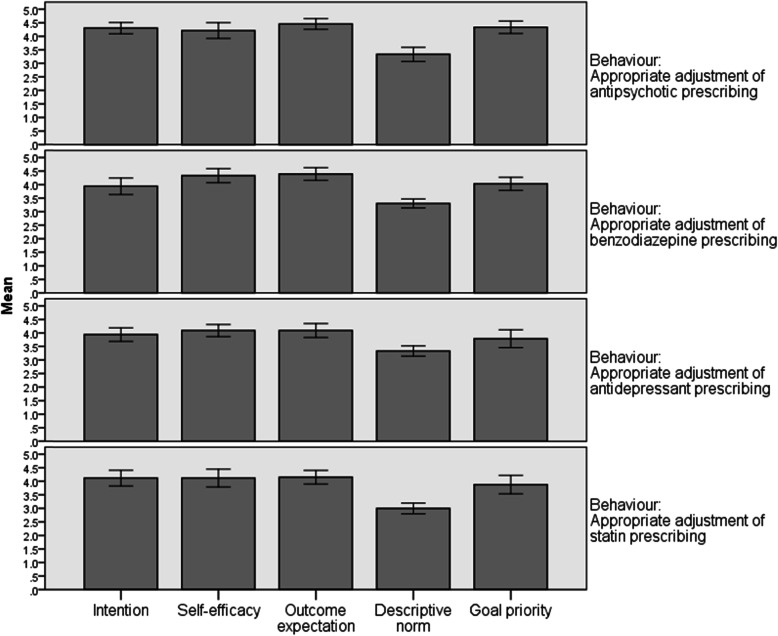
Descriptive characteristics for theory-based constructs assessed in relation to prescribing behaviour change (*n* = 33). Response scale: 1–5 Likert scale, strongly disagree–strongly agree (3 = neither agree nor disagree). Error bars represent 95% confidence intervals

Mean scores (Fig. 1) indicated that after receiving feedback, participants (i) intended to appropriately adjust their prescribing of all medication types (with the strongest intention for antipsychotics), (ii) were confident in their ability to appropriately adjust their prescribing of all medication types (with the greatest confidence for benzodiazepines), (iii) believed that appropriately adjusting prescribing of all medication types would avoid unnecessary risks to residents’ health (with the strongest belief for antipsychotics), and (iv) neither agreed nor disagreed that colleagues in other nursing homes in Ontario were appropriately adjusting their prescribing of all medication types. The broadest range in mean scores across behaviours occurred for goal priority (Fig. 1); while participants agreed that it was a priority for them to appropriately adjust prescribing of antipsychotics and benzodiazepines, they were less inclined to agree that appropriate adjustment of antidepressant and statin prescribing was a priority.

The multiple linear regression results showed the four hypothesised predictor constructs and two intervention factors explained between 84.4 and 24.1% of the variance in intention to appropriately adjust prescribing, depending on medication type. Amongst the four theoretical constructs tested as predictors of intention, goal priority was the only significant predictor of intention, and this was observed for all four medication types (Table 2); the greater the priority it was for physicians to appropriately adjust their prescribing, the stronger was their intention to appropriately adjust their prescribing.

**Table 2:** Results of multiple linear regression analyses predicting intention to appropriately adjust prescribing

	B	SE of B	*β*	*p*	95% CI for B	
Intention to appropriately adjust my prescribing of antipsychotics for my residents in my long-term care facility over the next month (*R*^2^ = .71)						
Self-efficacy	.15	.09	.22	.10	− .03	.34
Outcome expectations	− .01	.17	− .01	.95	− .36	.34
Descriptive norms	− .10	.11	− .13	.34	− .32	.12
Goal priority	.74	.14	.82	< .01	.45	1.03
Framing	− .15	.12	− .13	.23	− .40	.10
Comparator	− .26	.16	− .22	.11	− .58	.06
Constant	1.05	.58		.08	− .14	2.23
Intention to appropriately adjust my prescribing of benzodiazepines for my residents in my long-term care facility over the next month (*R*^2^ = .24)						
Self-efficacy	− .05	.23	− .04	.82	− .52	.42
Outcome expectations	.07	.28	.05	.80	− .50	.63
Descriptive norms	− .43	.37	− .23	.25	− 1.18	.32
Goal priority	.59	.25	.47	.03	.07	1.11
Framing	− .10	.30	− .06	.74	− .73	.52
Comparator	.05	.34	.03	.89	− .64	.74
Constant	2.90	1.66		.09	− .51	6.31
Intention to appropriately adjust my prescribing of antidepressants for my residents in my long-term care facility over the next month (*R*^2^ = 0.78)						
Self-efficacy	− .13	.11	− .12	.25	− .36	.10
Outcome expectations	.22	.13	.22	.10	− .04	.47
Descriptive norms	.10	.13	.08	.45	− .17	.38
Goal priority	.52	.10	.68	< .01	.31	.72
Framing	− .16	.13	− .12	.22	− .43	.11
Comparator	− .15	.13	− .10	.29	− .42	.13
Constant	1.45	.65		.03	.12	2.79
Intention to appropriately adjust my prescribing of statins for my residents in my long-term care facility over the next month (tracer outcome) (*R*^2^ = 0.84)						
Self-efficacy	.17	.09	.19	.06	− .01	.35
Outcome expectations	.02	.13	.02	.88	− .26	.29
Descriptive norms	.10	.12	.07	.44	− .15	.35
Goal priority	.66	.10	.78	< .01	.47	.86
Framing	− .01	.14	− .01	.98	− .28	.27
Comparator	− .22	.13	− .13	.12	− .49	.06
Constant	.59	.63		.36	− .71	1.89

*B* unstandardised regression coefficient, *β* standardised regression coefficient

### Qualitative interviews

#### Response rates and participant characteristics

Our initial response rate was lower than our minimum target sample size of 13, and so we modified our recruitment strategy. HQO sent email invitations to 14 new physicians who had signed up to receive their report, we reached out to our networks of physician contacts, and we asked interview participants to put us in contact with colleagues who may be interested. Despite this, we were unable to recruit 13 physicians. Five interviews were conducted, with all five physicians having taken part in the questionnaire study. Interviews had an average duration of 46 minutes (range = 36–64 min). Three participants were male and two were female. The physicians we spoke to typically spent only a small portion of their clinical time in the nursing home setting (aligning with findings in the quantitative data sample), and all practiced in more than one nursing home.

#### Prioritization of goals in nursing home care and in relation to audit and feedback

Four key themes were identified: (1) participants’ most prioritised goals in the nursing home setting were to improve quality of life and reduce the risk of falls, (2) government policy and media attention influenced participants’ prioritization of prescribing-related goals, (3) existing home-level quality improvement initiatives and routines had a stronger influence on participants’ prescribing than the A&F, and (4) addressing the antipsychotic prescribing indicator in the A&F report was a higher priority than addressing the benzodiazepine or specified central nervous system (CNS)-active medications indicators.

#### Theme 1: Participants’ most prioritised goals were to improve quality of life and reduce the risk of falls

When asked about their overall priorities in the nursing home setting, participants emphasised their focus on improving quality of life for their residents and reducing the risk of falls. These foci were the frame of reference for discussions relating to changing their prescribing behaviour. Physicians also mentioned priorities around controlling pain, preventing harm from medications, and reducing polypharmacy. The concept of achieving balance was discussed in relation to maintaining quality of life when prescribing antipsychotics.

‘I don’t mind seeing someone who’s a little bit more resistant to care as long as they can still have discussions with their family, rather than give them a ton of antipsychotics and they’re nice and quiet and they don’t bother anyone… but they don’t have any quality of life with their family, right?’ (P1)

#### Theme 2: Government policy and media attention influenced participants’ prioritization of prescribing-related goals

Our quantitative data indicated that appropriate adjustment of antipsychotic prescribing was a high priority. Three participants highlighted the influence of the Ontario Ministry of Health and Long-Term Care in driving this goal prioritization. Because the Ministry had prioritised appropriate adjustment of antipsychotic prescribing, this had become an individual priority.

‘About a year or two ago there was this big push by the Ministry, you are over using antipsychotic drugs. You should minimize it or try and stop them … So that’s when we started doing all this.’ (P4)

Two participants also mentioned pressure related to recent negative media coverage of antipsychotic prescribing in nursing homes, which was described as substantially impacting prescribing behaviour change.

‘I personally get very upset when I see those articles because we spend a lot of time trying to do the right thing… however the other side of the coin is that articles like that do stimulate us to take a look and make sure that we are doing appropriate prescribing.’ (P3)

‘My antipsychotics have gone down by almost half since that article in the Toronto Sun.’ (P1)

#### Theme 3: Existing home-level quality improvement initiatives and routines had a stronger influence on participants’ prescribing than the A&F

Participants highlighted that deprescribing initiatives or best practice routines were already in place in their facilities, and the behavioural focus of these initiatives often took precedence over those highlighted in the A&F. It was not always the case that participants mentioned a specific initiative, but rather their deprescribing behaviour seemed to be influenced by the routines or workflows associated with a specific facility. In some cases, the A&F complimented existing routines.

‘We’re active de-prescribers… honestly it’s standard of care in both my facilities amongst the physicians… and supported by the nursing staff. It’s just what we do.’ (P2)

‘We have what we call grand rounds where we meet every quarter with the nursing staff, nurse practitioner, the pharmacist, and the physicians. We sit down together and we develop best practices for our place... So all these ones that you have, like the benzodiazepines, we look at all those and we come up with best practices and then we try to implement that for our home.’ (P4)

#### Theme 4: Addressing the antipsychotic prescribing indicator in the A&F report was a higher priority than addressing the benzodiazepine or specified CNS-active medications indicators

The A&F report included data on three prescribing indicators: antipsychotic prescriptions for residents with dementia without psychosis, benzodiazepine prescriptions, and a metric showing the proportion of patients receiving three or more specific CNS-active medications (i.e. patients dispensed three or more medications from the following classes: antipsychotics, opioids, benzodiazepines, and antidepressants). Participants noted that all three indicators in the A&F were relevant to their over-arching goals outlined above, i.e. improving quality of life and reducing the risk of falls.

‘I think they’re all important because of what I said before. We’re trying to keep people safe and improve their quality of life… and if they’re over medicated, they will have a lesser quality of life than if they’re medicated properly.’ (P3)

Participants stated that of the three indicators, responding to the antipsychotic medication indicator by appropriately adjusting their prescribing was their highest priority (as indicated in the quantitative data). Appropriate adjustment of benzodiazepine prescribing was less prioritised, largely because participants perceived their benzodiazepine use to be low.

‘I probably prioritized the antipsychotics first, and then the 3 or more specified CNS active medications and then the benzos… because the Ministry is looking at the antipsychotics and sort of making a judgment about what kind of home you run depending on that report. In general, I find that I don’t use as many benzodiazepines… I just don’t use them that often and in general I don’t start them.’ (P5)

Prioritisation of the specified CNS-active medications indicator was rarely mentioned. One participant noted that this indicator included medications which were wide-ranging in relation to the potential for subsequent harm: they were therefore less likely to use this indicator to inform their clinical behaviour change efforts.

‘That one [i.e. the three or more specified CNS-active medications indicator in the A&F report] I don’t look so much at because I use a lot of antidepressants, and to me they are not as much of a danger as antipsychotics. I mean studies have shown that up to 80% of people in long-term care do have some form of depression… CNS active—a lot of things are CNS active, right?’ (P1)

## Discussion

### Summary of findings

Self-efficacy, outcome expectations, and descriptive norms did not predict physicians’ intention to appropriately adjust prescribing after receipt of A&F across any of the four prescribing behaviours (i.e. antipsychotics, benzodiazepines, antidepressants, and statins) in the nursing home setting. However, goal priority consistently predicted intention across all four behaviours: the greater the priority it was for physicians to appropriately adjust their prescribing, the stronger was their intention to appropriately adjust their prescribing. Qualitative data helped to explore which factors may have played a role in determining how physicians prioritised their goals, showing that appropriate adjustment of antipsychotic prescribing was a priority, influenced by recent negative media coverage of antipsychotic prescribing in nursing homes, the Ministry’s mandate to address antipsychotic prescribing, and because deprescribing initiatives or best practice routines were already in place in their homes.

### Implications for implementation interventions

To our knowledge, this is the first study to show that goal priority predicts healthcare providers’ intention. The evidence is further strengthened by the internal replication across four behaviours. That goal priority accounted for variability in intention to perform all of the prescribing behaviours, including those targeted and not targeted by the A&F, suggests that it may be a key component of physicians’ motivation relating to prescribing. This suggests that implementation interventions such as A&F which are explicitly designed to target goal prioritization have the potential to influence healthcare provider behaviour change via increased motivation.

In their study of health behaviour, Conner et al. [24] found that experimental manipulations to increase the priority of a specific goal further strengthened the relationship between intention and behaviour compared to a control condition. In the context of healthcare provider behaviour, A&F can be designed to influence prioritization of specific goals by directing attention and effort towards specific tasks relevant to achieving the goal, in line with Goal Setting Theory [1, 6]. Feedback reveals progress made towards goals and allows individuals to make adjustments to help them achieve the goal [1]. Of note, the manipulation of goal prioritization used by Conner et al. [24] incorporated active involvement of the participants, whereby they were required to write sentences about prioritising the behaviour of interest over other goals. Future A&F interventions may benefit from similar goal prioritization tasks specifically requiring input from the healthcare providers targeted.

Our qualitative findings provide further lessons for designing A&F to encourage prescribing behaviour change. It may be advantageous to design A&F to clearly align with external policy-driven targets. For example, the specific indicators included could be explicitly linked with relevant targets already prioritised. Since routines and workflows were described as exerting a strong influence over individual physicians’ prioritization, it may be beneficial to capitalise on this by developing and distributing nursing home-level A&F to drive team-level prioritization. This may be especially important in the context of nursing home care, since our demographic data indicate that physicians spend portions of their clinical time in different facilities and are therefore shifting between different routines and workflows and likely have significant constraints on their time in each facility. External targets, internal routines, and time pressures are key components of the context of nursing homes which can undermine the success of provider behaviour change initiatives, but interventions which involve multiple team members with a range of different roles can serve to unify the team and strengthen behaviour change efforts [32]. However, individual-level A&F data is generally more useful and actionable than team-level data [33], and so, if pursued, home-level A&F should supplement rather than replace individual A&F.

Finally, our qualitative results also suggest that if an indicator aligns with an area of lower concern from a physician’s perspective (i.e. benzodiazepine prescribing), changing behaviour in response to the indicator will generally not be prioritised. It is therefore important to align A&F with areas of concern from the physician’s perspective, where feasible and appropriate. This may involve removing indicators for which performance is indeed less concerning or moving them to a less prominent position in the A&F. This could help to minimise cognitive load associated with interpreting feedback [33] and avoid complacency that may transfer to other indicators. However, if the performance data do not align with physicians’ perceptions, additional intervention components to change perception of performance may be needed. It may be beneficial for A&F developers to be aware of potential discrepancies between healthcare providers’ perceptions of their own behaviour and objective behavioural data and address these in the A&F.

### Strengths and limitations

We have used theory-based quantitative measures and taken a multiple behaviour approach to investigate intention to appropriately adjust prescribing of multiple high-risk medications in nursing homes. Investigating multiple behaviours also allowed us to consider the relative priority of the different behaviours investigated. We incorporated qualitative methods to investigate contextual aspects of goal prioritization. However, a key limitation is that we have not included measures of behaviour. Nevertheless, the consistent relationship found between goal priority and intention highlights its importance as an aspect of motivation and aligns with previous work [24]. Our survey tool was brief, including one question per construct per behaviour, to minimise response burden. All participants responded to questions in the same order, and so we were unable to control for any potential order effects. Although we cannot rule out the possibility that social desirability bias impacted questionnaire responses, we took steps to mitigate this by making it clear in the instructions that responses would be kept anonymous and that study results would be published in aggregate and would not identify participants. We also acknowledged the similarity in item wording in the instructions and encouraged participants to provide their immediate responses. Differing relationships between the various constructs and intention suggests that items for those constructs were responded to differently. Future research could seek to investigate these issues, for example by counterbalancing the order of presentation or randomising inclusion of specific items. Our sample sizes were relatively small: this raises concerns regarding power in the quantitative analyses, although these were exploratory. Despite multiple and varied attempts to recruit physicians for interview, we were unable to recruit more than five physicians and have likely not achieved thematic saturation. However, our key themes map onto findings in the nursing home quality improvement literature, such as the influence of external policy and media reporting on behaviour change [32].

### Future research

Building on these findings, future research could investigate the extent to which goal priority is a consistent predictor of intention across multiple different behaviours of healthcare providers in varying roles. It is also important to investigate the relationship between goal priority and provider behaviour, ideally using objective behavioural data; if a direct relationship is found, as for other goal constructs [20], this would further inform efforts to target goal constructs in provider behaviour change interventions. Informed by the methods of Conner et al. [24], it would also be advantageous to conduct further experimental studies involving the manipulation of goal priority to determine causal relationships between goal priority, intention, and behaviour. It may be useful for manipulations to actively involve participants to encourage explicit consideration of priorities.

## Conclusions

Using a multiple behaviour approach, goal priority was found to predict nursing home physicians’ intention to adjust prescribing. The greater the priority it was for physicians to adjust their prescribing, the stronger was their intention. Physicians prioritised their goals on the basis of government policy, media attention, and facility-level initiatives/routines. Further work to determine the role of goal prioritization in healthcare provider behaviour would inform the development and evaluation of implementation interventions targeting goal priority to facilitate behaviour change.

## Supplementary information

**Additional file 1.** Good Reporting of A Mixed Methods Study (GRAMMS) Checklist.

**Additional file 2.** Qualitative interview questions.

## Data Availability

The datasets used during the current study are available from the corresponding author on reasonable request.

## Abbreviations

A&F: Audit and feedback
CFIR: Consolidated Framework for Implementation Research
CNS: Central nervous system
HQO: Health Quality Ontario
OHIL: Ontario Health Implementation Laboratory
OHRI: Ottawa Hospital Research Institute
WCH: Women’s College Hospital
