# Supplementary material for: A multiple-behaviour investigation of goal prioritisation in physicians receiving audit and feedback to address high-risk prescribing in nursing homes
Source: Implement Sci Commun. 2020 Feb 25;1:33. doi: 10.1186/s43058-020-00019-3 (PMC7427855; doi:10.1186/s43058-020-00019-3)
Supplement: Supplementary file 1 — Additional file 1. Good Reporting of A Mixed Methods Study (GRAMMS) Checklist. [file 43058_2020_19_MOESM1_ESM.docx]

**ADDITIONAL FILE 1: REPORTING CHECKLIST**

**Good Reporting of A Mixed Methods Study (GRAMMS)**

| **Guideline** | **Section: page** |
| --- | --- |
| Describe the justification for using a mixed methods approach to the research question | Design: p7 |
| Describe the design in terms of the purpose, priority and sequence of methods | Design: p7 |
| Describe each method in terms of sampling, data collection and analysis | Quantitative questionnaire: p8-9  Qualitative interviews: p9-10 |
| Describe where integration has occurred, how it has occurred and who has participated in it | Data analysis: p9-10 |
| Describe any limitation of one method associated with the present of the other method | Strengths and limitations: p18-19 |
| Describe any insights gained from mixing or integrating methods | Discussion: p16-18 |

*O'Cathain A, Murphy E, Nicholl J. The quality of mixed methods studies in health services research. J Health Serv Res Policy. 2008;13(2):92-98.*
