# Supplementary material for: A multiple-behaviour investigation of goal prioritisation in physicians receiving audit and feedback to address high-risk prescribing in nursing homes
Source: Implement Sci Commun. 2020 Feb 25;1:33. doi: 10.1186/s43058-020-00019-3 (PMC7427855; doi:10.1186/s43058-020-00019-3)
Supplement: Supplementary file 2 — Additional file 2. Qualitative interview questions. [file 43058_2020_19_MOESM2_ESM.docx]

**ADDITIONAL FILE 2: QUALITATIVE INTERVIEW QUESTIONS**

**Questions on report use and ideas for improvement**

VERIFY DATE OF REPORT CURRENTLY LOOKING AT

1. For starters, what are your overall views about your most recent HQO Long-Term Care Practice Report?
   - What was the best thing about the report?
   - What didn’t work so well/could be improved?
2. What parts of the report did you find the most useful? What made them useful?
   - E.g., Dashboard overview of all indicators, individual indicator pages, data about your facility, comparison to other MDs, change ideas

Great, thanks. The next few questions will help me get a better understanding of how you have used the report:

1. What motivated you to sign up to receive the reports, and to download and view this report?
   - E.g., General curiosity? Specific goals? Other relevant initiatives?
2. Can you describe where and how you accessed the report?
   - Where? Desktop/Laptop/Tablet/Phone? With whom?
   - How many different times did you look at the report?
3. How do you use the report in your long-term care facility?
   - Have you or will you use it to inform quality improvement initiatives? If so, how?
   - What would help you (better) use these reports to inform your clinical practice?
   - Do you involve others in your team/ use report in team context?
     1. PROBE professional roles if relevant: roles & responsibilities of team members (e.g. nurses & pharmacists) in relation to prescribing: could/should they also be targeted?
4. How easy is it to remember to use the report? What would make it easier?
5. Is using the report an automatic part of your job or is it something you need to stop and take time to think about?
   - Are the reports part of your routine or not?
6. Do you have any other views or opinions on improving the report for the future that have not already been covered?

**Questions on prioritization**

Thanks. I now have a few questions to try and help me understand how you consider your report alongside your goals and priorities in your facility.

1. So firstly, do you have specific goals that you try to meet when practicing in your long-term care facility? If so, what are these?
2. Did the data in your report influence these in any way?

You’ll recall that the report contains information related to three prescribing indicators, which are summarized on page 2: prescribing of benzodiazepines; prescribing of three or more specified CNS-active medications; and prescribing of antipsychotics for residents with dementia (without psychosis).

1. Given the data in your report, how important is it for you to adjust your prescribing of these drugs in the context of the goals and priorities you just described?
2. Did you review the information for all three indicators?
   - Why/why not?
3. Did you prioritise any of these three indicators over the others in relation to adjusting your prescribing?
4. To what extext do you feel that using the report to appropriately adjust prescribing is a priority of your long-term care facility?
5. What things influence your prescribing practices that are beyond your control?
   - Follow-up: How do these factors influence the way you interpret/use your report?

**Questions on intervention mechanisms of action**

Thanks. For the last part of our discussion, I have some specific questions to help me understand how the report might facilitate your decisions about prescribing (BENZODIAZEPINES) specifically, which you indicated was a priority for you.

(OR (IF BENZODIAZEPINE PRESCRIBING NOT A PRIORITY): three or more specified CNS-active medications/ prescribing of antipsychotics for residents with dementia (without psychosis) (DEPENDING ON PREFERENCE OF INTERVIEWEE))

(OR (IF NO INDICATORS PRIORITIZED): which would you prioritize if you had to pick one?)

Starting with the summary on page 2 of your report, you can see your (benzodiazepine) prescribing data for the residents in your long-term care facility, and you can also see the comparison of your prescribing data with that of other long-term care physicians in Ontario.

1. Can you tell me how this feedback changed your perception of your prescribing?
2. How did the feedback affect your intention to appropriately adjust your (benzodiazepine) prescribing?
   - IF INTENTION HIGH: is there anything else that influences your ability to act in relation to the feedback? GENERATING VS. TRANSLATING MOTIVATION: PROBE TO DETERMINE WHICH THE FEEDBACK DOES
3. In what way did the information influence your actual (benzodiazepine) prescribing?
4. What was the effect on your confidence in your ability to appropriately adjust your (benzodiazepine) prescribing?
   - IF CONFIDENCE HIGH: what barriers affect your confidence/what external factors get in your way?
5. Given this feedback, what do you think about the (benzodiazepine) prescribing practices of your colleagues in other long-term care facilities in Ontario?
   - IF RELEVANT TO CONVERSATION: if you could choose your comparator, which would you choose and why? Do you want a comparator at all?
6. Did the feedback influence your views on how appropriately adjusting your (benzodiazepine) prescribing affects your residents’ health?

For our final questions, I would like you to think about some examples of situations where you would prescribe (benzodiazepines).

1. When would you do this to improve a patient’s health, and when would you do this to prevent negative health outcomes?
2. Which of these seems to drive what you do more in this context (i.e. improving health vs. preventing negative health outcomes)?
3. Do you have any other thoughts on your (benzodiazepine) prescribing data, or on the comparison of your prescribing data with that of other long-term care physicians in Ontario, which have not been addressed already?

Those are all the questions I have for you. I appreciate the time and insight that you’ve given me today. Is there anything else related to this topic you would like to talk about that we haven’t covered?
